# Macular retinal thickness differs markedly in age-related macular degeneration driven by risk polymorphisms on chromosomes 1 and 10

**DOI:** 10.1038/s41598-020-78059-x

**Published:** 2020-12-03

**Authors:** Moussa A. Zouache, Alex Bennion, Jill L. Hageman, Christian Pappas, Burt T. Richards, Gregory S. Hageman

**Affiliations:** Steele Center for Translational Medicine, John A. Moran Eye Center, Department of Ophthalmology and Visual Sciences, University of Utah, Salt Lake City, 84132 UT USA

**Keywords:** Disease genetics, Macular degeneration, Clinical genetics, Genetics research

## Abstract

The two most common genetic contributors to age-related macular degeneration (AMD), a leading cause of irreversible vision loss worldwide, are variants associated with *CFH-CFHR5* on chromosome 1 (Chr1) and *ARMS2*/*HTRA1* on chromosome 10 (Chr10). We sought to determine if risk and protective variants associated with these two loci drive differences in macular retinal thickness prior and subsequent to the onset of clinically observable signs of AMD. We considered 299 individuals (547 eyes) homozygous for risk variants or haplotypes on Chr1 or Chr10 exclusively (*Chr1-risk* and *Chr10-risk*, respectively) or homozygous for a neutral haplotype (*Chr1-neu)*, for the protective I62 tagged haplotype (*Chr1-prot-I62)* or for the protection conferring *CFHR3/1* deletion haplotype (*Chr1-prot-del)* on Chr1 without any risk alleles on Chr10. Among eyes with no clinically observable signs of AMD, the deletion of *CFHR3/1*, which is strongly protective against this disease, is associated with significantly thicker retinas in the perifovea. When controlling for age, *Chr10-risk* eyes with early or intermediate AMD have thinner retinas as compared to eyes from the *Chr1-risk* group with similar disease severity. Our analysis indicates that this difference likely results from distinct biological and disease initiation and progression events associated with Chr1- and Chr10-directed AMD.

## Introduction

Age-related macular degeneration (AMD) is the leading cause of irreversible vision loss in the United States^1,2^ and is responsible for approximately 8% of global blindness^3^. Clinically, it is characterized at its early and intermediate stages by the presence of pigmentary abnormalities, drusen formation and pigment epithelial detachments in the fundus. Geographic atrophy (GA) and/or abnormal growth of choroidal and/or retinal vessels (neovascular AMD) in the fundus characterize late stage disease^4^. AMD susceptibility is determined by genetic predisposition and is modulated by risk factors that include age, smoking and diet^5,6^. The two most common genetic contributors to AMD are variants associated with a cluster of genes (complement factor H (*CFH*) – complement factor H-related (*CFHR*) 1 to 5) located on chromosome 1 (Chr1) and variants associated with age-related maculopathy susceptibility 2 (*ARMS2*) and high-temperature requirement factor A1 (*HTRA1*), two tightly-linked genes located on chromosome 10 (Chr10)^6–8^. Genome-wide association studies^9,10^ have identified thirty-two additional loci associated with AMD. These include other genes involved in the regulation of the complement system such as *C3*, *C2-CFB* and *CFI* and genes involved in lipid metabolism and extracellular matrix remodeling. However, these associations are minor when compared to genes located on Chr1 and Chr10 and only account for a small number of patients with AMD^6,10^.

Many aspects of the natural history of AMD including conversion to late stage disease^11–13^, prognoses^14,15^ and response to existing therapies for neovascular AMD^16^ differ between individuals with genetic risk on Chr1 or Chr10. This suggest that risk variants and haplotypes associated with the *CFH-CFHR5* locus and with *ARMS2*/*HTRA1* drive distinct biological and disease initiation and progression events. However, the specific contribution of genetic risk and protective variants and haplotypes on Chr1 and Chr10 to AMD clinical phenotypes and natural history remains to be fully elucidated. Improving our understanding of differences between Chr1- and Chr10-directed AMD and their respective associations with clinical phenotypes is key to improve patient care. It is also critical to design and apply adequate treatments against AMD and to identify meaningful clinical trial endpoints for future therapies.

All genetic risk on Chr10 is tagged by the variant rs10490924 (*ARMS2*)^17,18^ or single-nucleotide polymorphisms (SNPs) in strong linkage disequilibrium (LD) with it. Dissecting genetic risk within the *CFH-CFHR5* gene region has been challenging. Multiple studies have shown that the common missense variants Y402H (rs1061170) and I62V (rs800292) are strongly associated with risk and protection for AMD, respectively^7,19,20^. The *CFH* Y402H polymorphism alters the binding specificity of the *CFH* protein at the interface between retinal pigment epithelium^21^ and Bruch’s membrane^22^, which is the relevant location of AMD pathology, and for glycosaminoglycans^23,24^. The I62 *CFH* variant causes increased complement activity, which likely results in reduced complement activation and protection against AMD^25,26^. A common haplotype containing *CFHR3/1* deletion is protective against the development of AMD^27,28^. Haplotype analyses have shown that more than 90% of the genetic variability at the *CFH*-*CFHR5* locus could be explained by four common haplotypes that include the two SNPs rs1061170, rs800292 and the deletion of *CFHR3/1*^29^. One of these haplotypes confers an increased risk for AMD, two of them confer a protection against the development of disease, and one of them is present with similar frequencies in cases and controls, therefore it is neutral^26,29^. A recent GWAS study identified eight signals (4 common variants, 4 rare variants) within the *CFH* gene region independently associated with AMD^10^. One of the common variants is in complete LD with *CFH* Y402H, while the other three are non-coding and with unknown functional consequences. A haplotype analysis combining the four common variants, three rare variants and a SNP tagging the *CFHR3/1* deletion was recently performed^30^. The study identified an association between late AMD, risk haplotypes and elevated levels of circulating factor H-related protein 4.

Most current optical coherence tomography (OCT) systems offer the ability to measure and map the thickness of the retina and its distinct sublayers. The use of OCT machines is widespread, and retinal thickness has become a common and valuable tool to diagnose and monitor AMD. Previous studies have shown that individuals with AMD had thicker macular retinas as compared to healthy controls^31–33^. However, the pathways causing these changes remain to be identified. In this study, we sought to determine if macular retinal thickness varied among individuals with risk or protective polymorphisms on Chr1 or Chr10. Previous investigations reported that macular retinal thickness in individuals with risk alleles on Chr1 was either similar^34^ or smaller^35^ than that of subjects with risk alleles on Chr10. However, in both studies, AMD risk variants in patient groups were not mutually exclusive, and individuals with *CFH* protective variants or haplotypes were not considered. This considerably limited the ability of these investigations to identify variations in retinal thickness driven specifically by risk variants on Chr1 and Chr10. In addition, these studies did not account for factors known to modulate retinal thickness in genetically mixed populations. These factors include age^36,37^, AMD stage (early^38–40^ or intermediate^31–33,39,41,42^), smoking^36^, and the presence of reticular pseudo-drusen (RPD)^43^.

For this study, we considered individuals homozygous for a risk haplotype on Chr1 without any risk alleles on Chr10 (*Chr1-risk*), individuals homozygous for risk alleles on Chr10 without any risk alleles on Chr1 (*Chr10-risk*) and individuals homozygous for protective (*Chr1-prot-I62* and *Chr1-prot-del*) or neutral (*Chr1-neu*) haplotypes on Chr1 without any risk alleles on Chr10. This methodology allowed us to investigate the independent contribution of Chr1 and Chr10 to variations in retinal thickness associated with AMD. Genotype–phenotype associations identified in this study are therefore more likely to be exclusive to genetic risk and protective variants associated with either the *CFH-CFHR5* or *ARMS2*/*HTRA1* locus. By considering a large number of eyes with no clinically observable signs of AMD, we were able to determine if risk or protective polymorphisms on Chr1 or Chr10 drove differences in retinal thickness prior to the onset of clinical markers. We also estimated the specific contribution of age, gender, smoking and presence of RPD to mean macular retinal thickness.

## Results

### Baseline characteristics

**Figure 1:**
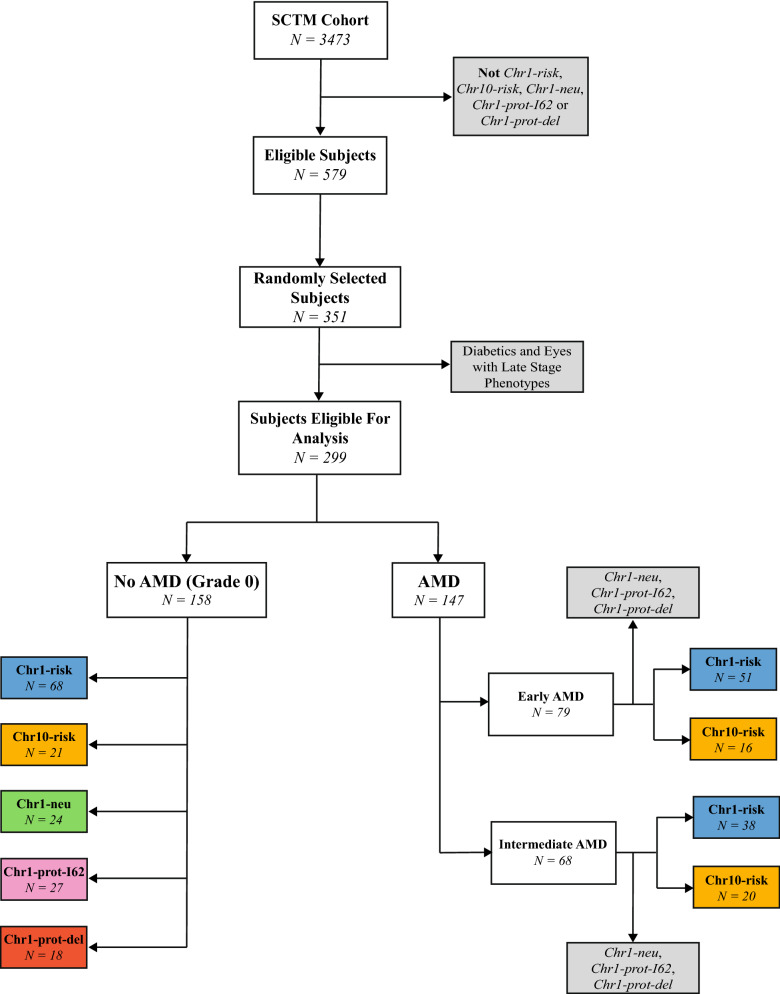
Diagram showing the methodology applied to select participants. The number of patients from AMD protective or neutral genetic groups with early or intermediate AMD was too low to assess differences in retinal thickness with the *Chr1-risk* and *Chr10-risk* groups. They were therefore excluded from the assessment of retinal thickness among subjects with AMD.

**Figure 2:**
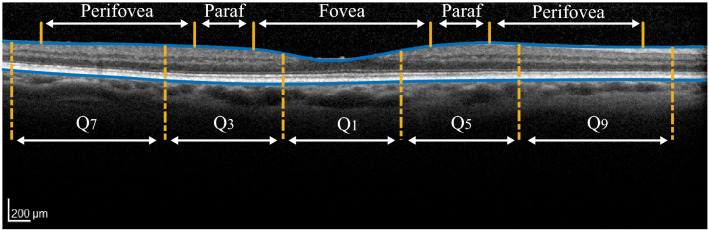
Schematic showing regions of the retina corresponding to sectors Q_1_, Q_3_, Q_5_ and Q_7_ of the ETDRS map on a B-scan, centred on the fovea. The B-scan was captured in an eye from the *Chr1-risk* group with a grade 0. The location of the histologically defined fovea, parafovea (here denoted *paraf*) and perifovea is also indicated.

Individuals included in this cross-sectional retrospective study were recruited between 2009 and 2017 at the Steele Center for Translational Medicine (SCTM), John A. Moran Eye Center, University of Utah, United States, as part of a case–control study into the genetic etiology of AMD. Written informed consent was obtained from all the participants present in this study. The SCTM cohort, which consisted of 3473 individuals, was stratified into genetic groups based on diplotypes on Chr1 and Chr10 (detailed in Supplementary Table S1). Diplotypes on Chr1 used haplotypes based on the amino acid altering variants Y402H (*rs1061170*) and I62V (*rs800292*)^7,19–25^ and the *CFHR3/1* deletion tagging SNP *rs12144939*^27,28^. Out of the four common haplotypes obtained using these SNPs, one is associated with an increased risk for AMD, two are protective, and one is present with frequencies similar in cases and controls and is labeled neutral^26^. Homozygous Chr10 risk and non-risk diplotypes are exclusively defined by *rs10490924* (*ARMS2*)^17,18^. Out of a total of 579 individuals having either mutually exclusive genetic risk on Chr1 or Chr10 (*Chr1-risk* and *Chr10-risk*, respectively) or the two same protective or neutral haplotypes on Chr1 without any risk alleles on Chr10 (*Chr1-neu*, *Chr1-prot-I62* and *Chr1-prot-del*), 351 were randomly selected for this study (see Fig. 1). Following exclusion of 44 diabetic individuals and eyes with late AMD, 1017 spectral domain optical coherence tomography (SD-OCT) volume scans were eligible for segmentation. Baseline demographics for the corresponding 299 participants (543 eyes) are presented in Table 1. Following grading (the grading scheme is detailed in Supplementary Table S2), eyes were classified into three groups based on AMD severity. These groups consisted of eyes with no clinically observable signs of AMD (grade 0), eyes with early AMD and eyes with intermediate AMD. The number of visits per eye varied between 1 and 18. Scans were recorded in both eyes for 242 (80%) patients. For each volume scan, the mean retinal thickness was computed for each sector of the 6 × 6 mm (1 mm, 3 mm, 6 mm) Early Treatment of Diabetic Retinopathy Study (ETDRS) map (see Fig. 2). Measurements made in the left (OS) and right (OD) eyes on the same visit correlated strongly ($0.87\le r\le 0.89$, *CI* [0.83; 0.92], $p<0.0001$ in all quadrants). The mean age for all participants was 74.3 ± 8.8 years old. There were no significant differences in the mean age of patients ($p=0.21$) or the proportion of males and females (${\mathrm{\rm X}}^{2}=0.96, p=0.97$) between genetic groups. The presence of RPD was recorded in 128 eyes of 75 participants (*Chr1-risk*: 47; *Chr10-risk*: 19; *Chr1-neu*: 6; *Chr1-prot-I62*: 2; *Chr1-prot-del*: 1). The proportion of individuals presenting RPD was smallest among neutral and protective groups (*Chr1-risk*: 30%; *Chr10-risk*: 36%; *Chr1-neu*: 16%; *Chr1-prot-I62*: 6%; *Chr1-prot-del*: 5%); this difference was statistically significant (${\mathrm{\rm X}}^{2}=18.7,p<0.001$). Eight patients (*Chr1-risk*: 2; *Chr10-risk*: 5; *Chr1-neu*: 1; *Chr1-prot-I62*: 0; *Chr1-prot-del*: 0) saw a change of AMD stage in one eye over the course of the visits recorded.

**Table 1:** Baseline demographics and AMD stages of subjects included in this study.

Demographic	Chr1-risk	Chr10-risk	Chr1-neu	Chr1-prot-I62	Chr1-prot-del	*p*-value for comparisons
**All participants**						
Subjects	155	52	37	34	21	
Age, mean (SD)	72.9 (9.6)	74.3 (7.9)	74.3 (7.8)	76.7 (9.8)	73.8 (7.6)	0.21^a^
Females	93	29	23	20	11	0.97^b^ (X^2^ = 0.96)
Males	62	23	14	14	10	
RPD	47	19	6	2	1	0.0008^b^ (X^2^ = 18.7)
Smokers	44	16	19	5	9	0.01^b^ (X^2^ = 13.24)
OD (with RPD)	146 (43)	43 (14)	33 (5)	31 (2)	21 (1)	–
OS (with RPD)	140 (42)	44 (15)	32 (4)	31 (2)	20 (0)	–
**AMD Grade 0**						
Subjects	68	21	24	27	18	
Mean age (SD)	70.3 (9.9)	70.5 (6.5)	71.6 (6.6)	76.8 (10.6)	75.9 (6.0)	0.009^c^
Females	46	8	15	15	9	0.22^b^ (X^2^ = 7.07)
Males	22	13	9	12	9	
RPD	3	3	1	2	0	–
Smokers	19	7	14	3	8	0.013^b^ (X^2^ = 14.4)
Eyes	130	37	45	50	36	
OD (with RPD)	63 (3)	18 (3)	23 (1)	25 (1)	18 (0)	–
OS (with RPD)	67 (5)	19 (2)	22 (0)	25 (2)	18 (0)	–
**Early AMD**						
Subjects	51	16	8	3	1	
Mean age (SD)	75.0 (9.7)	77.3 (8.6)	79.1 (7.2)	77.6 (6.0)	82.4 (-)	0.4^c,1^
Females	26	12	4	2	1	0.16^b,1^ (X^2^ = 1.97)
Males	25	4	4	1	0	
RPD	23	3	2	0	0	0.11^b,1^ (X^2^ = 2.5)
Smokers	14	5	6	1	0	> 0.9^b,1^ (X^2^ < 0.001)
Eyes	78	19	9	3	2	
OD (with RPD)	40 (19)	7 (0)	4 (1)	1 (0)	1 (0)	–
OS (with RPD)	38 (18)	12 (3)	5 (1)	2 (0)	1 (0)	–
**Intermediate AMD**						
Subjects	38	20	4	4	2	–
Mean age (SD)	77.9 (6.7)	78.6 (6.7)	83.1 (7.7)	74.2 (7.9)	60.7 (1.7)	0.71^c,1^
Females	22	16	4	3	1	0.16^b,1^ (X^2^ = 1.94)
Males	16	4	0	1	1	
RPD	21	13	3	1	1	0.66^b,1^ (X^2^ = 0.19)
Smokers	10	6	0	0	1	> 0.9^b,1^ (X^2^ < 0.001)
Eyes	59	28	7	5	3	–
OD (with RPD)	32 (18)	16 (11)	4 (3)	3 (1)	2 (1)	0.6^b,(1,2)^ (X^2^ = 0.27)
OS (with RPD)	27 (16)	12 (10)	3 (2)	2 (0)	1 (0)	0.3^b,(1,2)^ (X^2^ = 1.22)

^a^Kruskal-Wallis rank sum test.

^b^Chi-squared test.

^c^One-way ANOVA, significant pairwise comparison (Tukey): *Chr1-prot-I62*/*Chr1-risk*, *p* = *0.02.*

^1^Tests comparing *Chr1-risk* and *Chr10-ris*k groups only.

^2^Tests comparing the proportion of eyes with RPD only.

### Association between retinal thickness and gender, smoking and presence of RPD

When controlling for age, the retinas of females were on average thinner than those of males in all eyes regardless of AMD severity or genetic group. Differences following pairwise comparisons were only significant in Q_7_ for eyes with a grade 0 (${p}_{adj}=0.035$), in Q_4_ for eyes with early AMD (${p}_{adj}=0.01$) and at the fovea for eyes with intermediate AMD (${p}_{adj}<0.0001$). Smoking (${p}_{adj}>0.09$) or presence of RPD (${p}_{adj}>0.33$) did not significantly drive differences in macular retinal thickness regardless of genetic group or AMD severity.

### Macular retinal thickness in eyes with a grade 0 and no RPD

**Figure 3:**
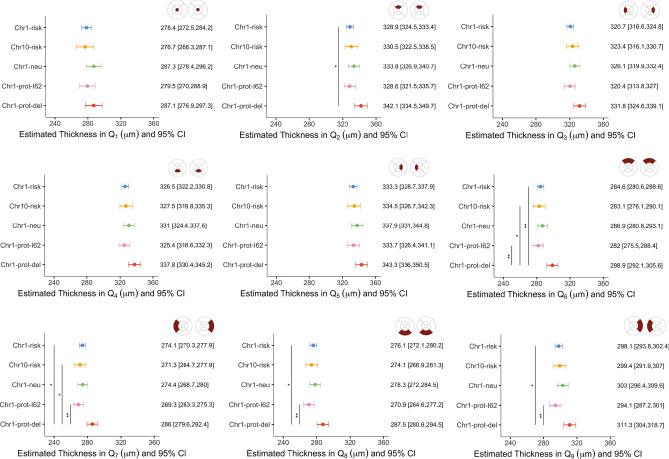
Estimated retinal thickness for eyes with a grade 0 and no RPD, by genetic group. Linear mixed-effect models included genetic group as the main independent variable and age, gender and smoking status as covariates. Marginal means were estimated from the mixed-effect models for each genetic group by averaging over the levels of gender and smoking status. The associated 95% confidence interval and significance levels adjusted for multiple comparison (***${p}_{adj}<0.001$; **${p}_{adj}<0.01$; *${p}_{adj}<0.05$) are shown.

Among eyes with a grade 0 and no RPD, age was the strongest predictor of retinal thickness in all quadrants ($p<0.001$) except from the fovea ($p=0.31$). Among all genetic groups macular retinal thickness declined by up to 8.9 µm (or 2.2% relative to baseline) per decade on average. No significant interactions between age and genetic group were found. When controlling for age, retinas from the *Chr1-prot-del* group were on average up to 6.3% thicker than those of individuals from the *Chr1-risk* (in Q_2_ and in the outer ring of the ETDRS map, ${p}_{adj}<0.03$), *Chr10-risk* (in Q_6_ and Q_7,_ ${p}_{adj}<0.02$), and *Chr1-prot-I62* (in the outer ring of the ETDRS map, ${p}_{adj}<0.004$) groups (see Fig. 3).

### Differences in macular retinal thickness among eyes with early or intermediate AMD

**Figure 4:**
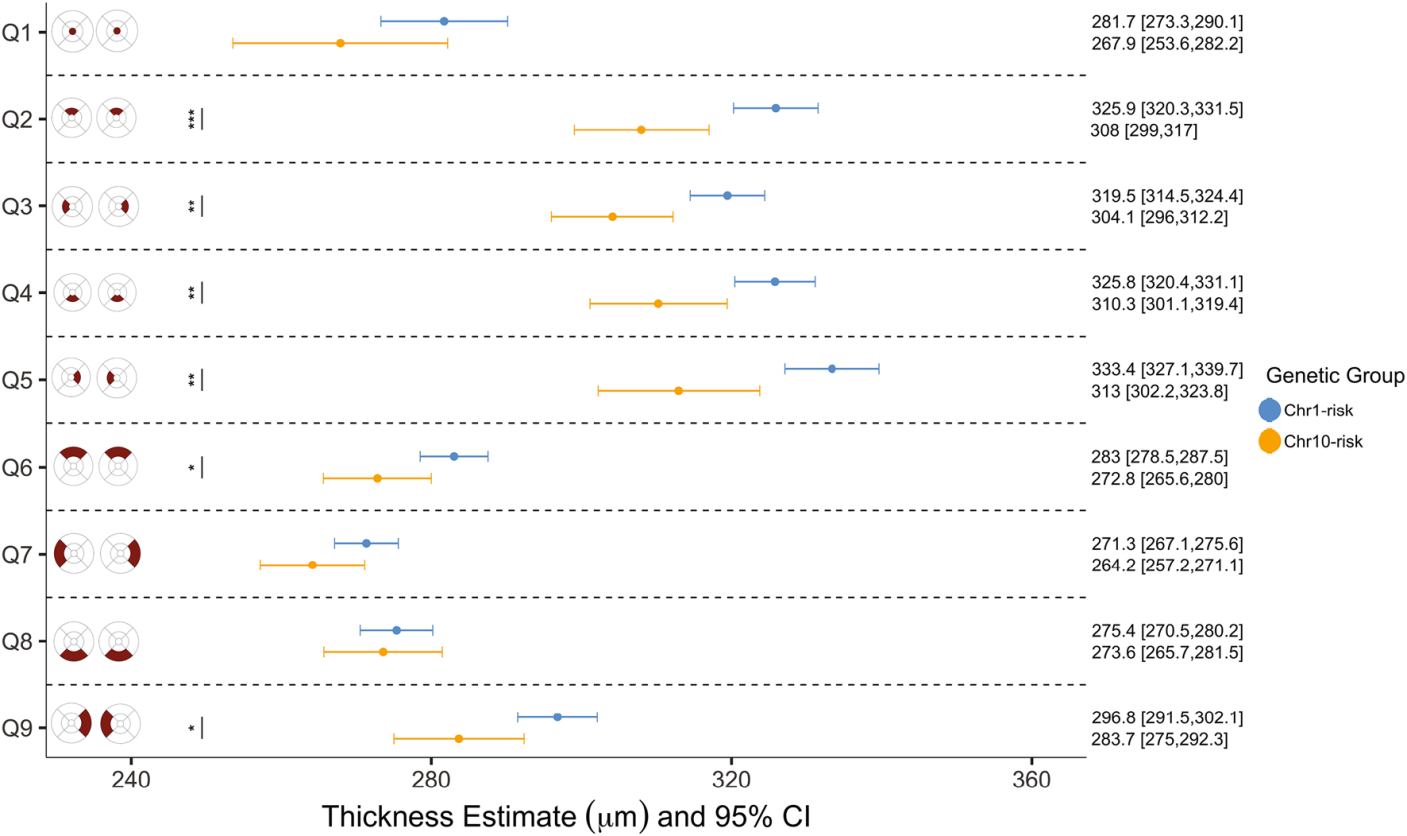
Estimates of retinal thickness in each quadrant of the ETDRS map in eyes with early AMD, by genetic group. Linear mixed-effect models included genetic group as the main independent variable and age, gender, smoking status and presence of RPD as covariates. Marginal means were estimated from the mixed-effect models for each genetic group by averaging over the levels of gender, smoking status and presence of RPD. The associated 95% confidence interval and significance levels adjusted for multiple comparison (***${p}_{adj}<0.001$; **${p}_{adj}<0.01$; *${p}_{adj}<0.05$) are shown.

**Figure 5:**
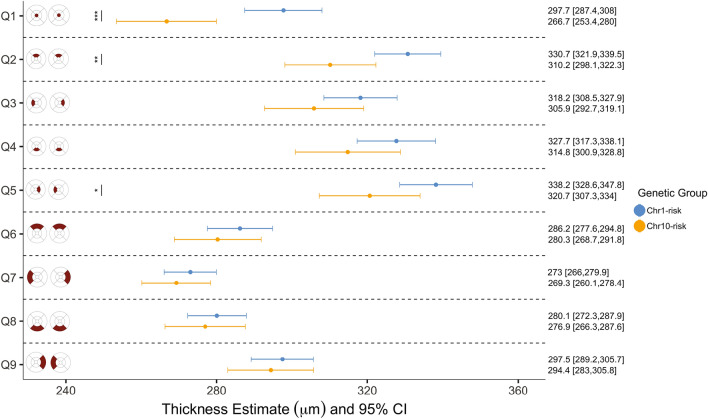
Estimates of retinal thickness in each quadrant of the ETDRS map in eyes with intermediate AMD, by genetic group. Linear mixed-effect models included genetic group as the main independent variable and age, gender, smoking status and presence of RPD as covariates. Marginal means were estimated from the mixed-effect models for each genetic group by averaging over the levels of gender, smoking status and presence of RPD. The associated 95% confidence interval and significance levels adjusted for multiple comparison (***${p}_{adj}<0.001$; **${p}_{adj}<0.01$; *${p}_{adj}<0.05$) are shown.

As expected, the number of patients from AMD protective or neutral genetic groups with early or intermediate AMD was low. As a result, only patients from the *Chr1-risk* and *Chr10-risk* groups were included when assessing associations between retinal thickness and AMD stage. When controlling for age, we found that retinas from the *Chr10-risk* group were thinner than those from the *Chr1-risk* group regardless of AMD stage. Among eyes with early AMD, differences between the two groups were significant in the inner quadrants of the ETDRS map (${p}_{adj}<0.01$), in Q_6_ and Q_9_ (${p}_{adj}<0.05$) but not in the fovea (${p}_{adj}=0.09$); see Fig. 4. Among eyes with intermediate AMD, differences between genetic groups were significant at the fovea (${p}_{adj}<0.0001$), in Q_2_ (${p}_{adj}=0.004$) and in Q_5_ (${p}_{adj}=0.023$); see Fig. 5.

Since there were no differences in macular retinal thickness between the *Chr1-risk* and *Chr10-risk* groups in eyes with a grade 0, we sought to determine if differences among eyes with early or intermediate AMD were driven by age. To do so, we extracted the change in retinal thickness with age (slope of the variation of retinal thickness with age) for each patient from linear mixed-effect models generated separately for each level of AMD severity. We then compared these slopes between genetic groups and between levels of AMD severity. We found that on average, age was associated with a reduction of the macular retinal thickness in all regions of the macula in both early and intermediate AMD regardless of genetic group. We also observed that the rate of retinal thinning varied spatially. It increased with disease severity in the central and inner ring of the ETDRS map in both *Chr1-risk* and *Chr10-risk* groups (see Supplementary Fig. S1). Retinal thinning was on average greater in the *Chr10-risk* group by 3.8 µm/decade in Q_2_ (${p}_{adj}=0.005$) and by 3.5 µm/decade in Q_5_ (${p}_{adj}=0.016$) in eyes with early AMD. It was greater by 5.3 µm/decade in Q_4_ (${p}_{adj}=0.03$) in eyes with intermediate AMD. These differences were however not systematic, and the rate of retinal thinning was actually greater in the *Chr10-risk* group than in the *Chr1-risk* group in some regions of the macula.

### Association between macular retinal thickness and AMD severity

Since variations in the rate of retinal thinning with age could not explain differences in retinal thickness between the *Chr1-risk* and *Chr10-risk* groups observed among eyes with early or intermediate AMD, we sought to assess if the pattern of change in retinal thickness associated with increasing AMD severity differed between the two groups. When controlling for age, we found that increasing AMD severity was associated with thicker retinas in eyes from the *Chr1-risk* group (see Supplementary Fig. S2). In this group, retinal thickness was greater in eyes with early AMD as compared to eyes with a grade 0 in all regions of the macula apart from the fovea. This difference was significant in the inner and outer inferior quadrants of the ETDRS map ($${p}_{adj}<0.003$$). The retina in eyes with intermediate AMD was thicker than that of eyes with a grade 0 in all quadrants, with differences significant in the inner and outer inferior quadrants ($${p}_{adj}<0.008$$), the fovea ($${p}_{adj}=0.0004)$$ and in Q_5_ ($${p}_{adj}=0.03)$$. The retina in eyes with intermediate AMD was thicker than that in eyes with early AMD in all quadrant but Q_9_; statistical significance for this difference was reached at the fovea ($${p}_{adj}<0.0001)$$. The association between retinal thickness and AMD severity was not as evident in the *Chr10-risk* group. In this group, the retina in eyes with early AMD was either within a similar range or thinner than that in eyes with a grade 0 across the macula. The retina was generally thicker in eyes with intermediate AMD as compared to eyes with a grade 0; although, statistical significance was not reached in any of the quadrants except for Q_8_ ($${p}_{adj}=0.044$$). The retina was significantly thicker in eyes with intermediate AMD as compared to eyes with early AMD only in Q_5_ and in the outer ring of the ETDRS map ($${p}_{adj}<0.049$$).

## Discussion

Among eyes with early or intermediate AMD, macular retinas from the *Chr10-risk* group are thinner that those from the *Chr1-risk* group at a similar stage of progression of the disease. This difference is not observed among eyes with a grade 0, which indicates that it appears subsequent to the onset of AMD clinical markers. Our analysis suggests that age is not the dominant driver of this difference. Retinal thickness decreases with age in eyes with early or intermediate AMD, with a pattern of decline that is similar in the *Chr1-risk* and *Chr10-risk* groups. Our results indicate that the main factor driving this difference is a general thickening of the retina associated with increasing AMD severity in the *Chr1-risk* group, which is not observed in the *Chr10-risk* group. This finding is likely to result from differences in the AMD disease process between Chr1- and Chr10-directed AMD.

Among eyes with a grade 0 and no RPD, retinal thickness decreases with age regardless of the region of the macula considered or genetic group. This is consistent with previous investigations of genetically mixed cohorts^34,44–46^. Previous reports found that foveal, perifoveal and peripheral macular thicknesses increase with age in younger individuals, but decrease in patients over 60^46^. This may explain the absence of correlation between thickness and age in cohorts with small sample sizes or with underrepresented age-groups^47,48^. Consistently with previous reports^34,48,49^, we found that the macular retina was thinner in females as compared to males; however, differences were only significant in one quadrant (Q_7_). Some have suggested that these differences were driven by thicker inner and outer nuclear layers in males^49^. While smoking has been identified as a modifiable risk factor for AMD^5,50–53^, we did not find any association between macular retinal thickness and smoking at baseline, which is also consistent with previous reports^54,55^. Once adjusted for age, baseline differences in retinal thickness among eyes with a grade 0 were driven by patients from the *Chr1-prot-del* group. These differences have not been reported before. Consistent with our findings, previous work found no associations between mean central, peripheral and total retinal thickness and the *CFH* Y402H, *CFH* I62V and *ARMS2* A69S variants in eyes without clinical signs of AMD^34^. A genome-wide association study (GWAS) found that the *CFH* and *ARMS2*/*HTRA1* loci were not associated with macular thickness in healthy individuals either^56^, which is consistent with our results in the fovea and the inner ring of the ETDRS map. However, because macular thickness was defined as an average within the outermost circle of the ETDRS map, it is likely that genes associated with local differences in thickness were more difficult to identify, which may explain why associations with the *CFHR3/1* locus were not found. The biological basis for differences between patients carrying the *CFHR*3/1 deletion and the *Chr1-risk*, *Chr10-risk*, and *Chr1-prot-I62* groups remains to be elucidated. Differences between carriers of the I62 tagged or *CFHR3/1* deletion haplotypes, which both confer protection against AMD, may indicate important differences in the mechanisms of protection associated with these two variants. Aging is associated with a decline of the ganglion cell layer^57,58^ and a steady decrease of the number of rod photoreceptors in the para- and perifovea^59^. The deletion of *CFHR3/1* might confer a protection against these processes at least in the perifovea, which might explain why retinas are comparatively thicker in this region.

Genetic risk on Chr1 was associated with a thickening of the retina as AMD severity increased from grade 0 to intermediate AMD. This thickening was largely absent among eyes from the *Chr10-risk* group. Changes in retinal thickness with AMD severity have mostly been investigated in populations with undefined genetic risk for this disease. A thickening of the RPE/BM complex^38,41^ and a thinning of the photoreceptor layer^38^ have been described in early AMD. Increased total retinal^31–33^ and RPE/BM^32,41,44^ thicknesses were reported in patients with intermediate AMD as compared to healthy controls. Other studies have shown that the outer retina was significantly thicker in the foveal region in individuals with intermediate AMD^41^. Our analysis suggests that the thickening of the retina with AMD severity described in previous studies is a feature of Chr1-directed AMD. Because our study was cross-sectional rather than longitudinal, it did not allow for the identification of a timeframe – defined either by age or stage of disease progression – over which differences between Chr1- and Chr10-directed AMD emerge. Future work will rely on longitudinal data to determine temporal variations in retinal thickness specific to each subset of this disease, and to identify specific retinal layers associated with the differences that we observe.

The functional significance of differences in macular retinal thickness between Chr1- and Chr10-directed AMD is yet to be elucidated. Functionally, increased retinal or RPE/BM thicknesses have been associated with visual field defects^39^ and a reduction of mesopic and scotopic sensitivities^31^. Too little is currently understood about the pathophysiology of Chr1- and Chr10-directed AMD and their differences to know if these features are specifically associated with *CFH* risk polymorphisms. Chr1-directed AMD is associated with high levels of complement activation at the RPE, Bruch’s membrane and choriocapillaris^60^, which may affect normal fluid transport across this interface and lead to fluid retention within the retinal tissue^61,62^. The vascular density and thickness of the choriocapillaris and the radius of choroidal arterioles and venules^63,64^ are key determinants of metabolite delivery to the outer retina and waste clearance from the subretinal space. Any variations in these features between Chr1- and Chr10-directed AMD may therefore partly explain the differences in retinal thickness that we observe. The phenotypic difference that we report between Chr1- and Chr10-directed AMD do point out to differences in pathology between these two subsets of AMD.

We found that the level of association between retinal thickness and age, gender and genetic group varied spatially across the retina. These differences may be due to spatial variations in the anatomy of the retina^65–68^ and choroid^64,69,70^, which might make certain areas of the eye more susceptible to AMD pathology. These geographical variations may also be partly due to the spatial selectivity of clinical markers of AMD. Drusen emergence is associated with a thinning of the retina^33,71^ and of all its layers^72^. Evidence suggest that the thickening of the retina occurs regardless of the presence of drusen in other retinal locations. The thickening of the retina and its outer nuclear layer observed in regions that are both close and distant from drusen led some to hypothesize that photoreceptor loss, which is characteristic of intermediate AMD^59,73^, occurs regardless of the spatial relationship with drusen^33^. By comparing regions with drusen and drusen-free areas at two separate time points, some have shown that an RPE layer thickening had a 71% sensitivity in predicting the location of drusen emergence^72^. However, the study did not control for age, which we found to be an important determinant of retinal thickness. Future work will focus on correlating changes in retinal thickness with the location of retinal lesions associated with AMD progression. We will also seek to correlate clinically observed changes in retinal thickness to pathological events occurring at the tissue level.

## Methods

### Participants

Written informed consent was obtained from all the participants present in this study. The study adhered to the tenets of the Declaration of Helsinki and was approved by the University of Utah Institutional Review Board. The SCTM cohort was stratified into genetic groups defined using risk or protective haplotypes on Chr1 and Chr10 and detailed in Supplementary Table S1. Diplotypes on Chr1 were defined on the basis of three tagging SNPs that included the risk-conferring *rs1061170* (*CFH 402*)^7,19,20,74^ and the protection-conferring *rs800292* (*CFH 62*)^7^ and *rs12144939* (*CFHR3/1*)^27,28^. Chr10 diplotypes were defined based on *rs10490924* (*ARMS2*)^17,18^. The *Chr1-risk* group consisted of patients homozygous for a risk haplotype on Chr1 who do not carry any risk alleles on Chr10. The two protected groups (*Chr1-prot-I62* and *Chr1-prot-del*) consisted of subjects homozygous for the protective I62 tagged haplotype or for the protection conferring *CFHR3/1* deletion haplotype without any risk alleles on Chr10, respectively. *Chr1-neu* was formed by patients homozygous for the neutral haplotype on Chr1^26,60,75^ without any risk alleles on Chr10. Patients homozygous for a risk allele on Chr10 with no risk alleles on Chr1 were grouped into the *Chr10-risk* group.

### Patient selection

A total of 579 individuals in the SCTM cohort met one of the following three selection criteria: homozygous risk haplotypes on Chr1 with no risk haplotypes on Chr10; homozygous risk haplotypes on Chr10 with no risk haplotypes on Chr1; identical protective or neutral haplotypes on Chr1 with no risk haplotypes on Chr10 (see Fig. 1). Out of these eligible participants, 57.5% belonged to the *Chr1-risk* group and 16.7% to the *Chr10-risk* group. Power calculations based on published estimates^76^ indicated that 351 subjects were sufficient to detect differences in macular retinal thickness of approximately 15 µm between genetic groups with an 80% power. We randomly selected 351 out of the 579 eligible participants for this study. For each patient selected all recorded visits were included. Eyes presenting with GA or neovascular AMD and patients with diabetes were excluded.

### Genotyping and haplotype analysis

Genotyping of the patients was described previously^60^. Briefly, following DNA extraction, the TaqMan platform (Applied Biosystems [ABI], Waltham, MA, USA) was used under the manufacturer’s recommended conditions to determine variant genotypes at the *CFH* and *ARMS2*/*HTRA1* loci (listed in Supplementary Table 1). The assay for *rs1061170* was made using ABI’s Custom TaqMan Assay Design Tool; all other SNP assays were predesigned. Samples were amplified using a PTC-225 Thermal Cycler (Madison James Research, Asheboro, NC, USA) and were analyzed on the Applied Biosystems 7900HT Fast Real-Time PCR System. Most likely haplotypes on Chr1 were extracted using the package *haplo.stats*^77^ and R^78^. The package uses an EM algorithm to analyze indirectly measured haplotypes and assumes that all subjects are unrelated.

### Imaging and grading

The same protocol was used to image all patients. For each visit a combined near-infrared reflectance (NIR) and spectral domain optical coherence tomography (SD-OCT) were performed using the Heidelberg Spectralis HRA + OCT (Heidelberg Engineering, Heidelberg, Germany). SD-OCT volumes consisted of 19 horizontal B-scans of the central 15° × 20° area with 10 frames averaged for each B-scan. The quality of the scans was assessed manually. The automatic scan alignment feature was used for follow-up scans. Every eye was graded for each visit independently by two independent investigators using fundus, NIR and SD-OCT volumes scans. Grading was based on the international classification of mutually exclusive stages of age-related maculopathy introduced by the Rotterdam Group^79^ (see Supplementary Table S2). Patients with no clinically observable signs of AMD or presenting drusen less than 63 µm in diameter were given a grade 0. The presence of RPD was identified using NIR and SD-OCT volume scans.

### Image segmentation and analysis

Semi-automated segmentations of three-dimensional SD-OCT volumes were performed using the HEYEX software (Heidelberg Engineering, Heidelberg, Germany). Retinal thickness was defined as the distance between Bruch’s membrane and the inner limiting membrane (see Fig. 2). Each B-scan was examined manually to ensure that the segmentation of these two retinal layers was accurate. When necessary, scans were re-centered prior to segmentation and generation of the retinal thickness. The mean retinal thickness in each sector of the 6 × 6 mm (1 mm, 3 mm, 6 mm) Early Treatment of Diabetic Retinopathy Study (ETDRS) map was computed by the HEYEX software. Labeling of the sectors from Q_1_ to Q_9_ is shown in Figs. 2 and 3. Following assessment and grading of all volume scans, sectors of the ETDRS map with macular holes, pigment epithelium detachment or vitreomacular traction were excluded from the analysis.

### Statistical analyses

The statistical analysis was performed using R^78^. The significance level was set at 0.05. Dependencies between variables including measurements made in the left and right eyes of the same patient were assessed using Bland–Altman plots, by plotting correlation curves and by calculating Pearson’s correlation coefficient, denoted *r*, and the associated confidence interval, denoted *CI*. Differences in retinal thickness between genetic groups and effects of covariates were investigated with linear mixed-effect models generated using the *lme4*^80^ package. These models included retinal thickness in one quadrant of the ETDRS map as the response variable, genetic group as the main independent variable and age, gender, presence of RPD, AMD severity and smoking status as covariates. Random effects of the form (1 + Age | Subject) were included to account for subject-specific variations in retinal thickness with age, multiple measurements over time and correlations between measurements made in the left and right eye. Interactions were investigated for predictors that had a strong effect when the sample size was sufficient. One model was generated for each region of the ETDRS map. Post-hoc analyses were performed independently for each quadrant of the ETDRS map to compute estimated marginal means (least-square means) and to perform pairwise comparisons between the different genetic groups using the *emmeans* package^81^. All *p*-values were adjusted for multiple comparisons using the Tukey method and were denoted *p*_*adj*_. Differences in the change in retinal thickness with age between genetic group were assessed by extracting the slope of the variation of thickness with age for each patient from linear mixed-effect models. Means of these slopes were compared using analysis of variance and by computing Tukey honest significant differences, which corrects for multiple comparisons.

## Supplementary information

Supplementary Information.
