# Author Correction: Macular retinal thickness differs markedly in age-related macular degeneration driven by risk polymorphisms on chromosomes 1 and 10

**DOI:** 10.1038/s41598-021-90883-3

**Published:** 2021-05-21

**Authors:** Moussa A. Zouache, Alex Bennion, Jill L. Hageman, Christian Pappas, Burt T. Richards, Gregory S. Hageman

**Affiliations:** Steele Center for Translational Medicine, John A. Moran Eye Center, Department of Ophthalmology and Visual Sciences, University of Utah, Salt Lake City, UT 84132 USA

Correction to: *Scientific Reports* 10.1038/s41598-020-78059-x, published online 03 December 2020

The original version of this Article contained a repeated error in gene labelling where,

“CFHR1/3”

now reads:

“CFHR3/1”

The labelling was correct in the Methods section and Supplementary Information file.

This error has now been corrected in the PDF and HTML versions of the Article.

