# Supplementary material for: Macular retinal thickness differs markedly in age-related macular degeneration driven by risk polymorphisms on chromosomes 1 and 10
Source: Sci Rep. 2020 Dec 3;10:21093. doi: 10.1038/s41598-020-78059-x (PMC7713215; doi:10.1038/s41598-020-78059-x)

Macular Retinal Thickness Differs Markedly in Age-related Macular Degeneration Driven by Risk Polymorphisms on Chromosomes 1 and 10

Moussa A. Zouache, Ph.D.[1,], Alex Bennion, B.S.[1], Jill L. Hageman, B.S.[1], Christian Pappas, B.S.[1], Burt T. Richards, Ph.D.[1] & Gregory S. Hageman, Ph.D[1,*]*

[1]Sharon Eccles Steele Center for Translational Medicine, John A. Moran Eye Center, Department of Ophthalmology and Visual Sciences, University of Utah, Salt Lake City, Utah, United States

* Corresponding authors: moussa.zouache@hsc.utah.edu, Phone: +1 (801) 213-2015
gregory.hageman@hsc.utah.edu, Phone: +1 (801) 213-2174

Supplementary Information

Supplementary Table S1: Genetic Diplotypes on Chromosomes 1 and 10 Employed to Stratify the Study Cohort.

Supplementary Table S2: Classification of Stages of AMD Employed.

Supplementary Figure S1: Box plots showing the variation of the rate of change in retinal thickness per decade (denoted β) with AMD severity, by genetic group.

Supplementary Figure S2: Estimates of retinal thickness in eyes with a grade 0 (including eyes with RPD), with early AMD or with intermediate AMD, by genetic group.

Supplementary Table S1: Diplotypes on Chromosomes 1 and 10 Employed to Stratify the Study Cohort.

Genetic Profile			Chr1			Chr10
Group	Chr1	Chr10	*CFH* 62 rs800292	*CFH* 402 rs1061170	*CFHR3/1* rs12144939	*ARMS2* rs10490924
**Chr1-risk**	Risk	Non-risk	GG	CC	GG	GG
**Chr10-risk**	Non-risk	Risk	AG/GG/AA	TT	GT/GG/TT	TT
**Chr1-neu**	Neutral	Non-risk	GG	TT	GG	GG
**Chr1-prot-I62**	Protection (I62)	Non-risk	AA	TT	GG	GG
**Chr1-prot-del**	Protection (*CFHR3/1* Deletion)	Non-risk	GG	TT	TT	GG

Supplementary Table S2: Classification of Stages of AMD Employed.

Grading was based on the international classification of mutually exclusive stages of age-related maculopathy introduced by the Rotterdam Group[79].

Findings on OCT	Stage of AMD
No observable sign of AMD<br>Small hard drusen (< 63µm in diameter)	Grade 0
Soft distinct drusen (≥ 63µm in diameter)	Early AMD
Isolated pigmentary changes only; no drusen (≥ 63µm in diameter)	
Soft indistinct drusen only (≥ 125µm in diameter)	
Soft distinct drusen (≥ 63µm in diameter) with pigmentary changes	
Soft indistinct drusen (≥ 125µm in diameter) with pigmentary changes	Intermediate AMD

Supplementary Figure S1: Box plots showing the variation of the rate of change in retinal thickness per decade (denoted $\boldsymbol{\beta}$) with AMD severity, by genetic group.

The change in retinal thickness per decade (slope of the variation of retinal thickness with age) was extracted for each patient from linear mixed-effect models generated separately for each level of AMD severity that included genetic group as the main independent variable and gender, age, smoking status and presence of RPD as covariates. Comparisons between genetic groups for each level of AMD severity and between levels of AMD severity were performed using analysis of variance and by generating Tukey honest significant differences. The 95% confidence interval and significance levels adjusted for multiple comparison (***: $p_{adj} < 0.001$; **: $p_{adj} < 0.01$; *: $p_{adj} < 0.05$) are shown.

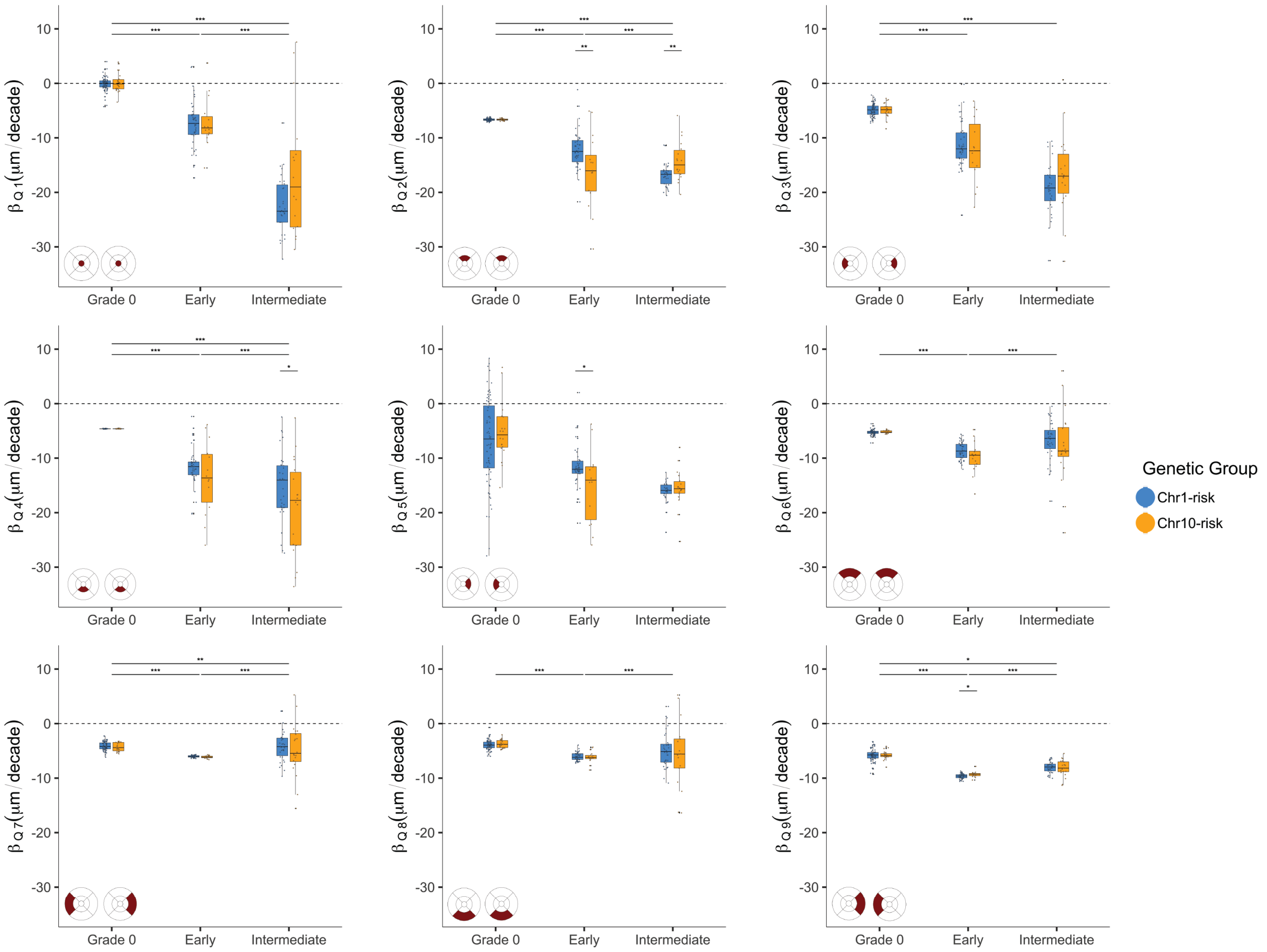

Supplementary Figure S2: Estimates of retinal thickness in eyes with a grade 0 (including eyes with RPD), with early AMD or with intermediate AMD, by genetic group.

Linear mixed-effect models that included AMD severity as the main independent variable and age, gender, smoking status and presence of RPD as covariates were generated separately for each genetic group. Marginal means were estimated from the mixed-effect models for each level of AMD severity by averaging over the levels of gender, smoking status and presence of RPD. The associated 95% confidence interval and significance levels adjusted for multiple comparison (***: $p_{adj} < 0.001$; **: $p_{adj} < 0.01$; *: $p_{adj} < 0.05$) are shown.

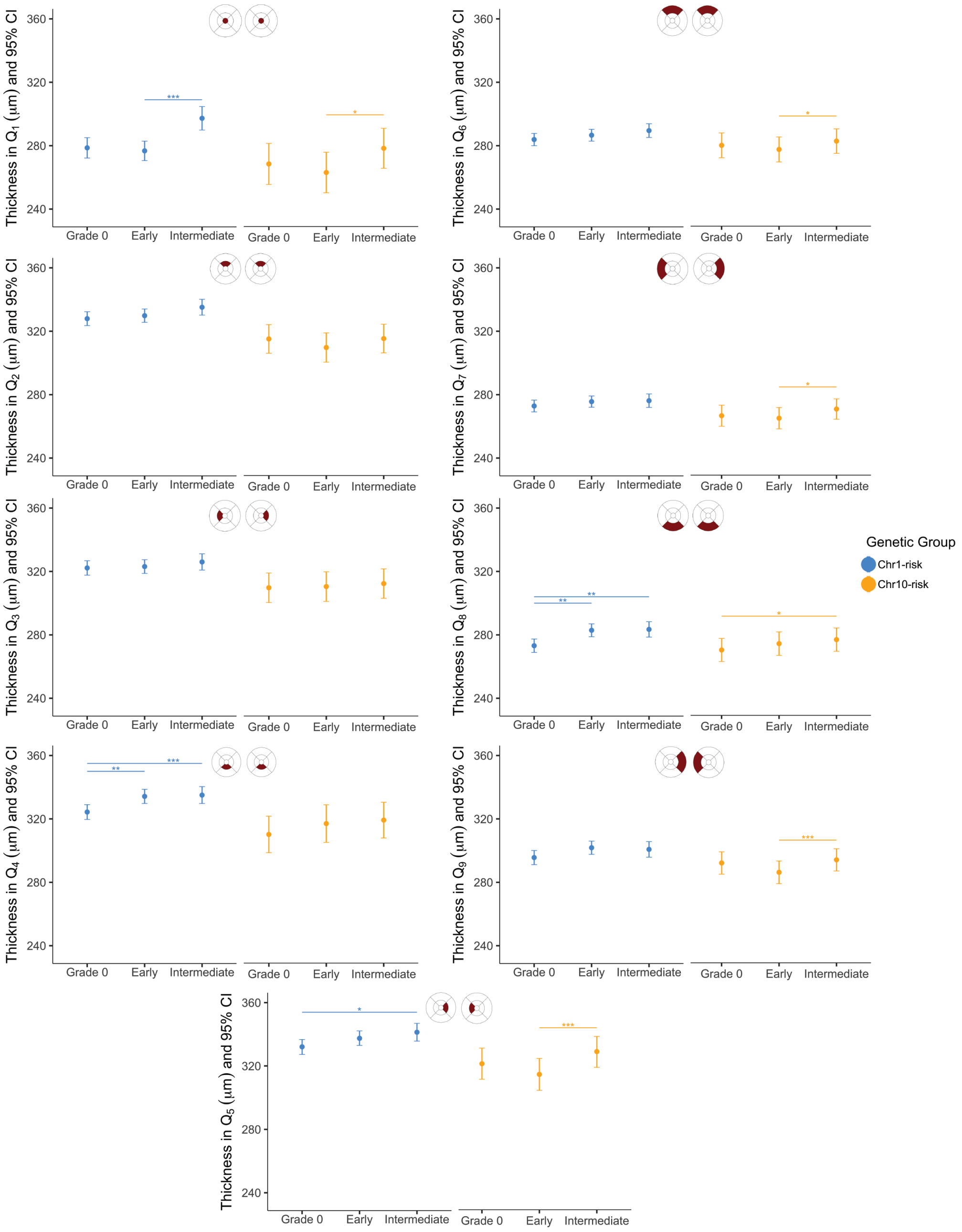

Supplement: Supplementary file 1 — Supplementary Information. [file 41598_2020_78059_MOESM1_ESM.pdf]
